# Protein Intake in NCAA Division 1 Soccer Players: Assessment of Daily Amounts, Distribution Patterns, and Leucine Levels as a Quality Indicator

**DOI:** 10.3390/sports11020045

**Published:** 2023-02-14

**Authors:** Jun Kwon, Morgan M. Nishisaka, Alexandra F. McGrath, Aleksandra S. Kristo, Angelos K. Sikalidis, Scott K. Reaves

**Affiliations:** 1 Nutrition Program, Department of Food Science and Nutrition, California Polytechnic State University, San Luis Obispo, CA 93407, USA; 2 Cal Poly Personalized Nutrition Research Group, California Polytechnic State University, San Luis Obispo, CA 93407, USA; 3 Center for Health Research, California Polytechnic State University, San Luis Obispo, CA 94307, USA

**Keywords:** sports nutrition, dietary protein, collegiate athletes, NCAA, soccer

## Abstract

Dietary protein is required to support recovery and adaptation following exercise training. While prior research demonstrates that many athletes meet total daily protein needs, intake seems to be predominantly skewed toward the evening meal. An even distribution of protein doses of ≥0.24 g/kg BW consumed throughout the course of a day is theorized to confer greater skeletal muscle anabolism outcomes compared to a skewed pattern of intake. Protein quality is also an important dietary consideration for athletes, with the amino acid leucine seemingly serving as the primary driver of the postprandial anabolic response. The present study investigates protein consumption characteristics among a cohort of NCAA D1 soccer players and evaluates differences between male and female athletes. Athletes were instructed to complete 3-day food diaries, which were subsequently analyzed and compared to UEFA expert group-issued nutrition guidelines for soccer players. Breakfast, lunch, and dinner accounted for 81.4% of the total daily dietary protein intake. Most athletes (77.8%) ingested optimum amounts of protein at dinner but not at breakfast (11.1%) or lunch (47.2%). In addition, statistically significant sex-based differences in daily dietary protein intake, meal-specific protein amounts, and protein quality measures were detected. Findings indicate suboptimal dietary protein intake practices among the collegiate soccer athletes.

## 1. Introduction

Soccer is a predominantly aerobic sport that also includes frequent bouts of explosive activity, such as short sprints, tackles, duel play, and jumps, which depend on anaerobic energy production [1,2]. As soccer training elicits substantial musculoskeletal stress, disruption, and damage, dietary protein is needed to support recovery and adaptation [1,3,4,5,6]. Moreover, dietary protein is a key variable for lean body mass (LBM) accrual, which in turn can confer performance benefits to the soccer player by improving strength and power output [3,7].

A consensus has been formed by major sports nutrition organizations that athletes seeking to optimize body composition and performance should consume 1.2–2.0 g protein/kg BW/day; higher intakes may be advantageous during certain conditions, e.g., periods of energy restriction [8,9]. The consensus report authored by FIFA/FIFA-Medical Assessment Research Center (F-MARC) in 2006 specifies that a maximum of 1.2–1.6 protein/kg BW/day may be needed by soccer athletes, though stating that the evidence of this is “not clear or universal” [10]. By comparison, more recently issued soccer-specific sports nutrition guidance by an expert group commissioned by the Union of European Football Associations (UEFA) recommends protein intakes in the range of 1.6–2.2 g/kg BW/day [3], in concert with accumulating evidence that these intakes maximally augment the skeletal muscle adaptive response to exercise training [11,12]. 

In addition to consuming sufficient total daily protein, emphasis has been placed on distributing adequate dietary protein amounts evenly over the course of a day across multiple meals in order to maximize muscle protein synthesis (MPS). Acute feeding studies utilizing amino acid tracer methodologies indicate a saturable dose-response relationship between dietary protein and postprandial MPS, such that maximal MPS is achieved with a serving of ~0.24–0.4 g protein/kg BW/meal at rest and after resistance training [8,13,14,15]. Protein servings within this range likely contain the requisite amount of the amino acid leucine (~2–3 g) needed to fully augment MPS [3,8,16]. While all amino acids serve as ‘building blocks’ of muscle proteins, leucine has emerged as the most potent driver of the MPS response to protein-containing meals [17,18,19]. To that end, the leucine content of foods has been suggested to be an important measure of protein quality, as sufficient doses of it are needed on a per-meal basis to maximally stimulate MPS [20,21,22,23]. Lower protein doses, corresponding to <2 g leucine, may lead to an attenuated postprandial MPS response, whereas amounts beyond this range do not further amplify MPS; instead, excess amino acids are shunted toward energy production [16,24]. Longer term studies have reported favorable effects on 24-hour MPS [25] as well as strength- and body composition-related outcomes [26] following conditions in which sufficient protein is consumed on a per-meal basis and evenly distributed across the day, as compared to disproportionate distribution patterns. In accordance with these findings, the UEFA expert group advises the consumption of at least ~0.4 g protein/kg BW containing ~2.5 g leucine over 3–4 discrete meals throughout the day and ~0.4–0.5 g protein/kg BW prior to nighttime sleep [3]. 

There is a paucity of research addressing female athlete-specific dietary protein requirements, and it is unclear if protein needs are altered as a function of menstrual cycle phase or hormonal contraceptive usage [27,28]. The most comprehensive systematic review on this topic by Mercer et al. [28] reported that the protein requirements of pre-menopausal female athletes appear to be “within the mid- to upper range of current sports nutrition guidelines.” Nevertheless, the limited inclusion of active female participants in protein nutrition and metabolism research precludes the ability to establish sex-specific dietary protein intake recommendations at present. In concordance with the current state of research, the UEFA expert panel does not issue separate protein intake targets on the basis of sex [3]. Interestingly, however, literature reviews of observational studies evaluating habitual protein consumption amounts in soccer players highlight that male players generally consume more protein than their female counterparts (1.9 vs. ~1.45 g protein/kg BW/day, respectively) [29,30].

Dietary protein intakes and distribution patterns have been previously characterized among various athletic cohorts, such as elite Dutch and Canadian athletes, semi-professional to professional rugby union players in Australia and New Zealand, and English soccer players [31,32,33,34,35]. While these studies are heterogenous with respect to methodologies and participant demographics, they are generally consistent in reporting that protein intakes are predominantly skewed toward the evening. To date, however, the practices regarding the intake of dietary protein on a meal-per-meal basis have not been thoroughly and fully characterized in National Collegiate Athletic Association Division 1 (NCAA D1) soccer players to the best of our knowledge. 

The primary aims of the present study were to observe and quantify habitual dietary protein intake patterns among a sample of collegiate soccer players in an NCAA D1 program during the beginning of the season. The total amount of protein consumed daily, distribution of protein across the main meals, and protein quality measures were evaluated in relation to UEFA sports nutrition consensus guidelines for elite soccer players. Differences between males and females in protein consumption characteristics were also explored.

## 2. Materials and Methods

### 2.1. Participants

A total of 54 NCAA Division I soccer players (males, n = 30, females, n = 24) were evaluated for eligibility. Of those, 36 soccer players (males, n = 13, females, n = 23) returned food records that were of sufficient detail to analyze and thus be included in the study (see participant characteristics in Table 1). All student-athletes were informed of the procedures, benefits, and risks of their involvement in the study and provided written informed consent prior to their participation. Participants were made aware that their involvement in the study was voluntary. The study was reviewed and approved by the Institutional Review Board for Human Subjects Research of California Polytechnic State University, San Luis Obispo, and was in accordance with the declaration of Helsinki pertaining to human studies (IRB No: 2018-274).

### 2.2. Dietary Data Collection and Analyses

Following an educational sports nutrition presentation led by qualified nutrition personnel, participants were provided standardized 3-day food diary forms along with thorough verbal and written instructions to accurately record all foods and beverages consumed. The athletes were instructed to record in detail the type, amount, and method of preparation of the food eaten. To account for variations in dietary intake across the week, participants completed the 3-day food diary forms to include two weekdays and one weekend day. In some cases, 2-day food records were accepted in cases where the athletes failed to return 3-day records, constituting ~30% of food records. Participants’ body weight measurements were determined using a manual scale at the time of dietary data collection. Individuals were asked to arrive in a fasted state and refrain from wearing heavy clothing.

Data from food diaries were then manually entered and analyzed by trained sports nutrition research assistants using the Food Processor III Nutrition Analysis Software (version 11.11.32, ESHA Nutrition Research, Salem, OR, USA) to evaluate habitual dietary intakes, as previously described [36]. Three-day averages of total energy, total dietary protein, and per-meal dietary protein amounts were calculated for each participant. Protein values were expressed as absolute terms, relative to body weight (g/kg BW), and as a percentage of total daily energy intake. Meal-specific protein intakes (breakfast, lunch, and dinner periods) were also expressed in both absolute and relative values. Daily relative protein intakes were compared with the UEFA-issued protein recommendations of 1.6–2.2 g/kg BW/day. Likewise, protein intake at each meal was compared with the per-meal dose of ≥0.4 g protein/kg BW advised by the UEFA expert group. As the UEFA nutrition guidelines emphasize the relevance of leucine, the actual-to-ideal ratio of dietary leucine was generated for each participant using the Protein Quality Report function of the nutrition analysis software. The ratio is based on the National Academy of Medicine’s amino acid scoring pattern of 55 mg leucine/g protein for all age groups ≥1 year of age [37]. Results were then averaged for men and women, and sex-based comparisons were conducted.

### 2.3. Statistical Analyses

Statistical analyses were conducted using JMP Pro software (version 16.0; SAS Institute Inc.; JMP Statistical Discovery, LLC, Cary, NC, USA). The normality of distributions was tested using the Shapiro–Wilk test for each response variable. Deviations from normality were observed for some response variables. The non-parametric Wilcoxon signed-rank test for non-normally distributed variables and independent Student’s *t*-test for normally distributed variables were used to assess sex differences in total daily energy and protein intakes. To perform within- and between-group comparisons of meal-specific relative protein amounts, a 2-way repeated measures ANOVA was used with Tukey’s test for post-hoc analyses of multiple comparisons. Statistical significance was set a priori at α = 0.05, two-sided. Data are presented as means ± standard deviation (SD) or percentages.

## 3. Results

### 3.1. Participant Characteristics

The characteristics of the 36 student-athletes included in the analyses are displayed in Table 1.

### 3.2. Dietary Energy and Protein Intake

Total daily energy and protein intakes are presented in Table 2. Men displayed higher intakes of total energy (2714 ± 529 vs. 1907 ± 447 kcal/day, *p* < 0.001), absolute protein (151.8 ± 39.4 vs. 87.7 ± 23.9 g/day, *p* < 0.001), and relative daily protein amounts (expressed as g/kg BW) (2.08 ± 0.51 vs. 1.38 ± 0.35 g/kg BW/day, *p* < 0.001), and protein as a percentage of total energy intake (22.77 ± 5.99 vs. 18.53 ± 3.57%, *p* = 0.03), as compared to women (Table 2). The minimum protein intake of 1.6 g/kg BW/day, as advised by UEFA guidelines, was met, or exceeded by 10/13 (77%) and 8/23 (35%) of men and women, respectively (Table 3). Further, 7/36 (19.4%) of athletes, all of whom were men, exceeded the upper limit recommendation of 2.2 g protein/kg BW/day. Daily protein intakes ranged from 1.29 to 2.86 g/kg BW for men and 0.86 to 2.02 g/kg BW for women. 

### 3.3. Dietary Protein Distribution

**Figure 1:**
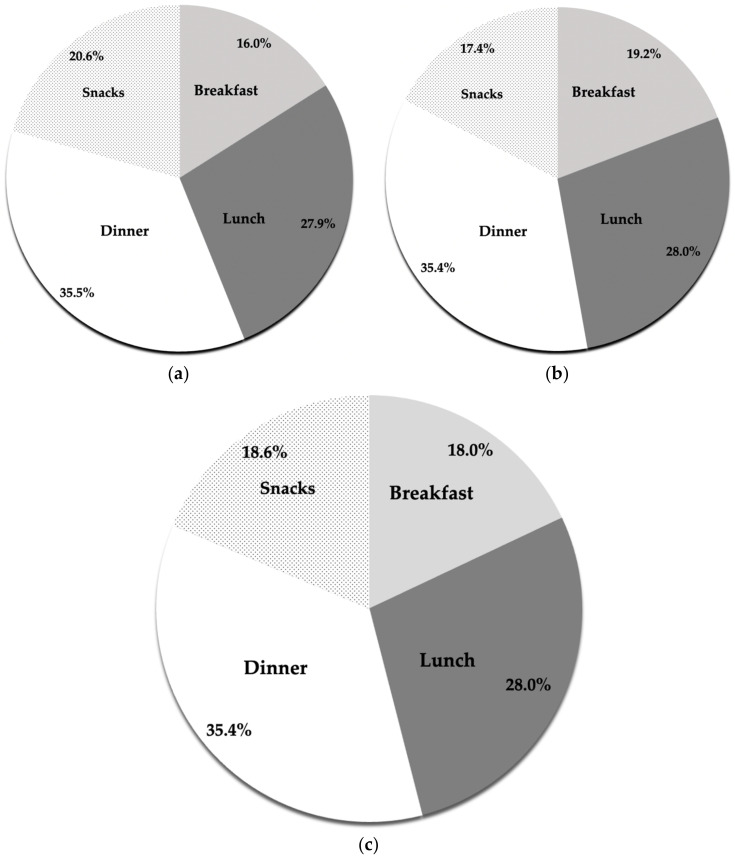
Dietary protein distribution across meals: (**a**) male soccer players, (**b**) female soccer players, (**c**) all athletes. The pie charts describe the respective contribution of the main meals and snacks toward total daily protein.

Dietary protein intakes were disproportionately distributed across the main meals. Breakfast, lunch, and dinner meals contributed, on average, 18, 28, and 35.4% of total daily protein intake, respectively, demonstrating that most protein (81.4%) was consumed during the three main meals (Figure 1). Broken down by sex, 16, 27.9, and 35.5% of the protein in men and 19.2, 28, and 35.4% in women were consumed at breakfast, lunch, and dinner, respectively.

The per-meal dietary protein target of ≥0.4 g/kg BW was met by 4/36 (11.1%) participants at breakfast, 17 (47.2%) at lunch, and 28 (77.8%) at dinner (Table 3). On all three reported eating occasions, fewer female players met the per-meal protein target. Dinner represented the highest protein-containing meal for 27 (75%) of the participants. Conversely, 2.8% and 22.2% of athletes reported breakfast and lunch to be the highest protein-containing meals, respectively. 

On average, protein intakes were below optimum levels for both male (0.33 ± 0.23) and female (0.26 ± 0.11 g/kg BW) participants at breakfast (Table 4). Men consumed sufficient mean per-meal protein amounts at lunch (0.60 ± 0.39 g/kg BW), while women fell slightly below recommendations (0.38 ± 0.16 g/kg BW). Mean protein intakes at dinner were adequate for men and women (0.71 ± 0.26, 0.49 ± 0.16 g/kg BW, respectively).

**Figure 2:**
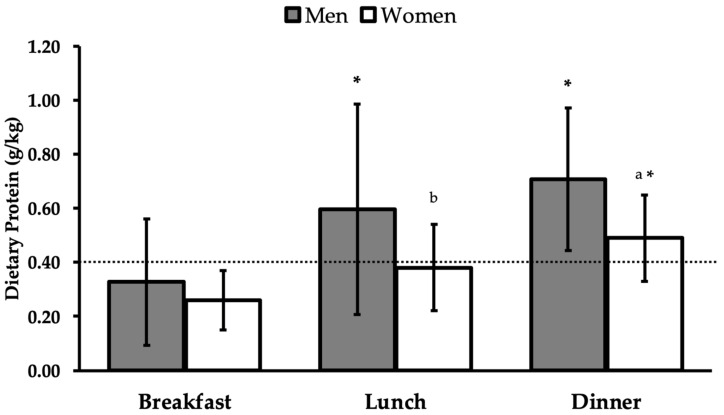
Mean protein intakes relative to body weight at breakfast, lunch, and dinner meals in men and women. The dashed line represents the minimum per-meal threshold of 0.4 g protein/kg BW. Data are presented as means ± SD. * Significantly different from breakfast (*p* < 0.05). ^a^ Significantly different from men at the same meal. ^b^ Statistical tendency to differ from men at the same meal.

Within- and between-group comparisons of meal-specific relative protein intakes are presented in Figure 2. Mean relative protein intakes at dinner were significantly greater when compared with breakfast for both men and women (*p* < 0.001, *p* = 0.007, respectively). Men also consumed significantly more protein at lunch compared with breakfast (*p* = 0.02), but no apparent differences in breakfast and lunch protein intakes were noted for women (*p* = 0.36). When compared with women, men had significantly higher relative protein intakes at dinner (*p* = 0.048) and tended to consume more protein at lunch (*p* = 0.06). 

### 3.4. Dietary Protein Quality

**Figure 3:**
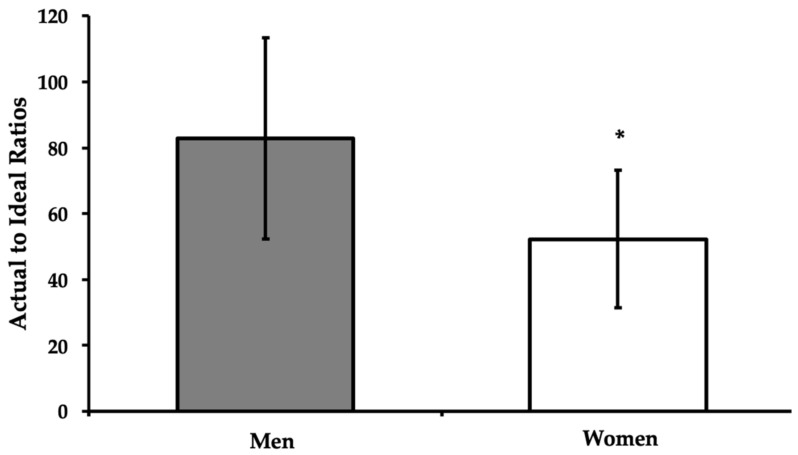
Mean actual-to-ideal intake ratios of leucine. Data are presented as means ± SD. * Significantly different from men (*p* < 0.05).

The Protein Quality Report compared the actual vs. ideal ratio of leucine in the diet between men and women. On average, male participants were found to have a significantly higher actual-to-ideal leucine proportion than females (82.82 ± 30.46% vs. 52.2 ± 20.86%, *p* = 0.005) (Figure 3).

## 4. Discussion

The aims of the present study were to observe and quantify the daily intake, distribution, and quality measures of dietary protein—all of which represent important nutritional determinants of skeletal muscle anabolism—in a sample of NCAA D1 soccer players. Potential sex differences in these variables were also explored. Consistent with previous findings, total daily energy and protein intakes were significantly higher in men than in women. The distribution of dietary protein was uneven across meals such that intakes skewed toward meals later in the day. 

It has been suggested that the overall amount of dietary protein consumed daily appears to be the principal nutritional determinant of skeletal muscle anabolism. Though it was originally proposed that the Recommended Dietary Allowance (RDA) of 0.8 g protein/kg BW/day is sufficient for athletes, recent meta-analyses suggest that optimum adaptation occurs with intakes of ~1.6 g protein/kg BW/day when paired with appropriate exercise training, though intakes upwards of ~2.2 g protein/kg BW/day may be warranted in certain cases [11,12,37,38,39]. Consistent with the most recent evidence, the UEFA expert panel advises intakes of 1.6–2.2 g protein/kg BW/day for soccer players [3]. All participants in the present study met the RDA for dietary protein; however, when compared against UEFA protein guidelines, only 35% of women achieved such intakes whereas 77% of men met or exceeded recommendations. Complementing these findings, mean daily protein intakes were within UEFA recommendations for men (2.08 ± 0.51) but not for women (1.38 ± 0.35 g/kg BW/day). Nonetheless, both men and women, on average, were within the National Academy of Medicine’s Acceptable Macronutrient Distribution Range (AMDR), which is defined as “a range of intakes for a particular energy source that is associated with reduced risk of chronic diseases while providing adequate intakes of essential nutrients,” of 10–35% of total energy intake as protein [37]. Of note, the habitual daily protein intake amounts of the men’s and women’s soccer players observed in the present cohort are in line with previous findings in soccer players [29,30].

Given that sufficient leucine, i.e., 2–3 g, is needed to augment MPS, the leucine content of foods has been suggested to be a critical measure of protein quality [20,21,22]. Hence, a focus was placed on the leucine content as a proxy for dietary protein quality. We report that, on average, men had higher actual-to-ideal intake ratios of leucine compared to women, which suggests greater relative amounts of high-quality, animal-derived protein-containing foods present in the diet; however, protein sources were not directly evaluated in this study. The actual-to-ideal leucine intake ratio may serve as a practicable, albeit crude, aggregate measure of the overall quality of protein sources in a diet; to our knowledge, no other study has used this ratio for similar purposes.

In addition to protein quality and total daily quantity, the within-day distribution of dietary protein across meals has also emerged as an important variable of consideration for athletes. A pivotal paper in this field is a highly cited meta-analysis by Moore et al. [24], which pooled results from six studies that, utilizing stable-isotope amino acid methodologies, measured acute MPS responses to single doses of high-quality, readily digestible animal-derived protein sources at rest. The authors described a saturable per-meal protein threshold dose of ~0.24 g/kg BW (corresponding to ~2–3 g leucine) that results in maximum postprandial MPS in young adults. Similar findings have been reported in studies evaluating postprandial MPS responses to dietary protein following resistance training bouts, though MPS rates are further augmented as exercise enhances the sensitivity of muscle to the anabolic properties of protein [13,16,40]. Factoring in possible interindividual variability and acknowledging “real world” mixed-macronutrient meals that likely include lower quality protein sources and food matrices that modify protein digestion, absorption, and aminoacidemia, subsequent publications have advised intakes of ~0.4–0.5 g protein/kg BW/meal for athletes [15,41,42], including soccer players [3]. Taken together, distributing protein doses evenly across meals to reach the target threshold of ≥0.4 g protein/kg BW multiple times across the day may induce greater cumulative MPS responses as compared to disproportionate distributions on a total daily protein intake-adjusted basis. Nonetheless, observations of dietary habits among athletes, as well as general population adults in the United States and Europe, have consistently indicated that protein intakes are skewed toward later meals, with intakes at breakfast and lunch generally insufficient to maximally stimulate MPS [33,43,44,45]. Herein, we found that ~85% of participants reported meeting the protein threshold for MPS at dinner, but only ~15% and ~54% did so at breakfast and lunch, respectively. Moreover, both male and female participants displayed significantly higher relative protein intakes at dinner in comparison to breakfast. Finally, ~81% of the total daily protein was consumed at the main meals, with breakfast, lunch, and dinner meals contributing 18, 28, and 35% to total daily protein intake, respectively. From a practical perspective, these findings imply that athletes could be advised to include additional servings of protein-rich foods, e.g., 1–2 more eggs, at breakfast as a means to meet per-meal protein recommendations. 

While there is broad agreement that a balanced protein distribution is likely to confer a greater anabolic response as compared to an uneven distribution pattern, it is important to acknowledge that not all studies have provided universal support to this idea. In this regard, Hudson et al. [46] presented examples of observational and interventional studies that failed to detect positive relationships between balanced protein distribution patterns and muscle-related outcomes. The authors conveyed that equivocal results may be explained by inadequate protein quantity/quality at meals that do not account for differences between whole-food animal and plant-protein sources from isolated intact proteins often used in dose-response studies. As such, there is a need for future work to establish relative saturable doses of protein-rich whole foods. Discrepancies in findings may also be explained by substantial heterogeneity among studies with regard to total protein quantities, age, state of energy balance, and the presence of exercise training stimuli. A separate systematic review reported a positive association between even protein distributions and muscle mass but not with muscle strength [47]. Although there are several unresolved questions, given that acute- and longer-duration clinical studies [13,25,26], as well as several nutrition consensus statements authored by credible groups [3,8,9], endorse the consumption of protein doses meeting the MPS threshold in evenly divided meals throughout the day, it seems prudent for collegiate athletes seeking to maximize training adaptations to adopt these practices. Biological plausibility in favor of such practice supports the idea for a bolus provision of amino acids as raw materials as well as energy and signaling agents to maximize MPS via optimized availability of resources and signals.

It was unsurprising that breakfast contributed the least toward total daily protein and that only a minority of participants reported consuming recommended per-meal protein amounts at breakfast. These results mirror previous observational studies of athletes, perhaps reflecting at least in part the diurnal rhythm of ghrelin, the principal hormone in the regulation of hunger [32,33,48]. In addition to biological rhythms, it has been suggested that age, ethnicity, geographical factors, and education levels also influence breakfast intake patterns [49]. Recommendations to increase protein intake at breakfast require careful contextualization. Health-promoting protein-rich foods, such as eggs, yogurts, and lean meats, should be preferentially emphasized. On the other hand, consumption of processed meats, including those commonly consumed at breakfast., e.g., bacon and sausages, has been consistently associated with increased risk for chronic disease; thus, their use should be minimized [50]. 

The UEFA expert group endorses a ‘food first’ philosophy but acknowledges that dietary supplements and sports foods may be useful in instances where this approach is not feasible [3]. Accordingly, protein supplements may be advised for athletes who are otherwise unable to obtain sufficient amounts of protein-rich foods at breakfast or other meals. While whey protein supplements are commonly recommended due to their high leucine content, digestibility, and wide commercial availability [3], there is growing interest in vegan and vegetarian diets among athletes [51]. To that end, recent work by Maykish et al. [52] suggests that almond protein powder may represent a functional alternative to whey protein. Indeed, to increase the likelihood of adherence, dietary programs should maximize user feasibility by tailoring to the personal preferences and tolerances of the athlete to the extent possible. Qualified nutrition personnel can offer effective, practical nutrition education and consultations aimed at improving athletes’ protein-rich food consumption patterns. 

To date, most of the protein metabolism studies in the literature have been conducted on male participants. Though a considerable degree of uncertainty surrounds the topic due to limited data, there is no compelling evidence to issue sex-specific dietary recommendations for total daily protein intake, distribution, and quality of protein at present [27,28]. In general, however, the literature suggests that male soccer players may typically consume more protein than female players do [29,30], a pattern likely explained by the fact that muscle mass accretion usually represents a higher priority goal among male athletes [53,54]. Along similar lines, this study demonstrated that total daily protein quantities—expressed in absolute amounts, on a body weight-adjusted basis, and as a proportion of total energy consumption—were all significantly lower in women as compared to men. Additionally, on a per-meal basis, men consumed significantly more protein at dinner and tended to consume more at lunch than women did. Mean protein intakes were above 0.4 g/kg BW in two of the three main meals for men but only in one of three meals for women. Further contextualizing these observations, roughly 9, 44, and 74% of female athletes achieved sufficient per-meal protein intakes compared with 15, 54, and 85% of males who did so at breakfast, lunch, and dinner, respectively. Despite no discernable sex-dependent differences in dietary protein needs, female soccer players exhibited suboptimal levels of intake on both a daily and meal-to-meal basis and may thus be at greater risk for impaired skeletal muscle adaptive responses to training. While this study offers evidence for sex-related differences in protein intake quantities and patterns, an important caveat is that there were more women than men included in the analyses. Consequently, men may be underrepresented in this study, thus calling for future work on this topic to employ a larger sample size of men. 

Studies utilizing the doubly labeled water technique, widely considered the gold standard method for energy expenditure assessments, have reported typical mean daily energy expenditures of ~3500 and ~2700 kcal/day in men and women’s soccer players, respectively [3,55,56,57,58]. Though not a primary focus, we quantified and reported the energy intakes of athletes in the present study. Strikingly, participants displayed mean energy intakes (men, 2714 ± 529, women, 1907 ± 447 kcal/day) that were markedly lower than estimated expenditures, thereby suggesting a state of net energy deficit. The UEFA expert group advises in-season carbohydrate intakes between 6 and 8 g/kg BW/day and fat intakes of 20–35% of total daily energy intake. In line with previous findings in soccer players [58], our findings suggest it is likely that athletes in our study may consistently be consuming less than adequate carbohydrate and fat, and their overall low-calorie intake may result in dietary protein being used for energy, hence not available for addition and maintenance of lean body mass. Echoing these findings, our group has also observed inadequate energy and carbohydrate intake in collegiate men’s basketball players [36]. While the most obvious solution is to seek and implement dietary programming approaches to increase caloric intake, it is important to acknowledge that the importance of dietary protein is further magnified in this scenario. Previous work has shown that during high physical demand energy deficit conditions, higher protein diets containing ≥2.2 g/kg BW/day may mitigate the loss of LBM in athletic populations [59,60] while, in some cases, actually promoting LBM gain [38]. In this regard, the UEFA expert group states that higher protein intakes, upwards of ~2.4 g/kg BW/day, may be recommended during high physical demand energy deficit periods [3].

In addition to male soccer players being potentially underrepresented in the study, another limitation of the study design is that the timing of the main meals in relation to one another was not assessed. The MPS response to protein-containing meals is acute and transient, exhibiting a ‘muscle-full’ effect wherein an additional protein bolus consumed during this period fails to exert additive effects on MPS [61]. Accordingly, to maximize daily MPS, some guidelines advise athletes to organize protein intakes so that sufficient quantities are consumed every 3–5 h [8,9]. Further, consuming dietary protein in close temporal proximity to the training session has been strongly advised on the grounds that it may exert particularly pronounced effects on adaptations to exercise [56]. In this regard, the UEFA expert panel writes that protein supplements may be advisable in doses of 0.3–0.4 g/kg BW in the postexercise period [3]. Nonetheless, because protein timing was not directly addressed in the present study, it is difficult to ascertain the extent to which the athletes adhered to these recommendations. In addition, relative protein intakes at snacking periods were not analyzed as individual meals/events (i.e., breakfast, lunch, and dinner); snacks were thus combined in our analyses and represented roughly 20% of total daily protein intake. Conceivably, snacks may have consisted of sufficient protein quantities to maximally stimulate MPS. Future studies should address these areas. 

Likely as a function of the substantial time and effort required to maintain detailed food records amid demanding class and training schedules, many athletes failed to return completed food diaries. As a result, the study is limited by its relatively modest sample size. In order to expand on these findings and improve generalizability, there is a need for further research on dietary protein consumption characteristics utilizing a larger sample of collegiate soccer athletes. There are other notable and unique challenges in conducting field-based nutrition research on athletes in free-living conditions. Specifically, it is also important to note that college athletes lead highly demanding schedules since they are not only athletes but also college students and do not have the support and ability to focus on their athletic tasks to the extent that professional athletes typically afford.

There are other notable and unique challenges in conducting field-based nutrition research on athletes in free-living conditions. While methods relying on the self-reporting of dietary intake, including the 3-day food record used in the present study, are widely employed by nutrition researchers and practitioners, there are several limitations to this approach. The high degree of burden posed on the athletes to record dietary information with accuracy may explain the results of a systematic review authored by Capling et al. [62], who found that mean self-reported energy intake was underestimated by 19% (range, 0.4–36%). Some investigations have noted substantial bias toward underreporting at higher energy expenditures has also been reported, i.e., the higher the expenditure, the greater the extent of energy intake under-estimation [62]. In view of these limitations, some investigators may be inclined to consider utilizing longer recording durations which better account for day-to-day variations and provide a more accurate representation of habitual intakes [62]. A noteworthy downside to this approach, nonetheless, is the possibility for higher rates of attrition. Strategies to strengthen the methodologic rigor and certainty of dietary assessments include the usage of food records in conjunction with other methods of dietary data collection, as well as with biomarkers of nutritional adequacy and energy expenditure measurements, though these approaches are admittedly more costly and invasive. Future studies should adopt a combined dietary assessment approach and evaluate the relationship between protein intake characteristics, body composition, and performance.

## 5. Conclusions

Although habitual dietary protein consumption patterns among soccer players have been previously characterized in the peer-reviewed literature, to the best of our knowledge, this is the first report to quantitively describe total daily intake, distribution, and quality of protein among NCAA D1 soccer players and explore potential sex-related differences. Moreover, while several studies have reported mean daily protein intakes of collegiate athletes in relation to existing recommendations, few have addressed the proportion of the sample athletes meeting daily and per-meal recommendations. By reporting on these data, this study offers a more complete view of habitual dietary practices in relation to protein consumption patterns. Notwithstanding the limitations noted above, we found that, as a whole, male athletes appeared to have slightly more favorable eating habits in relation to the intake, distribution patterns, and quality of dietary protein. Nonetheless, both male and female athletes exhibited protein consumption patterns that deviated markedly from soccer-specific nutrition guidelines authored by the UEFA-commissioned expert panel. Of note, a high percentage of student-athletes failed to meet the protein threshold for MPS at the breakfast meal, followed by lunch and dinner meals. Crucially, these results highlight that a sizeable proportion of student-athletes may be at risk for suboptimal musculoskeletal recovery and adaptation, ultimately impairing sports performance. Finally, in agreement with recent findings of inadequate energy, protein, and carbohydrate intake in collegiate male basketball players, the present findings emphasize the need for trained sports nutrition professionals who can provide appropriate nutrition education and counseling for collegiate athletes regarding optimal dietary protein feeding practices. Although a ‘food first’ approach is preferred, dietary protein supplements may be advised for some athletes, particularly when considering breakfast.

## Figures and Tables

**Table 1:** Descriptive characteristics of participants ^1^.

	Men	Women
Sample size (n)	13	23
Age (years)	19.7 ± 1.3	19.4 ± 1.5
Height (cm)	180.3 ± 7.8	167.0 ± 5.4
Body weight (kg)	72.9 ± 7.4	63.5 ± 5.5
BMI	22.4 ± 1.4	22.7 ± 1.3

^1^ Data are presented as means ± SD.

**Table 2:** Daily energy and protein intakes ^1^.

	Men	Women
Total energy (kcal)	2714 ± 529	1907 ± 447 *
Protein (g)	151.8 ± 39.4	87.7 ± 23.9 *
Protein (g/kg BW)	2.08 ± 0.51	1.38 ± 0.35 *
Protein (% total kcal)	22.77 ± 5.99	18.53 ± 3.57 *

^1^ Data are presented as means ± SD. * Statistically significant differences between men and women (*p* < 0.05).

**Table 3:** Participants meeting dietary protein recommendations ^1^.

	% Consuming ≥1.6 g/kg BW/Day	% Consuming ≥0.4 g/kg BW at Breakfast	% Consuming ≥0.4 g/kg BW at Lunch	% Consuming ≥0.4 g/kg BW at Dinner
Men	77.0%	15.4%	53.8%	84.6%
Women	35.0%	8.7%	43.5%	73.9%
Total	50.0%	11.1%	47.2%	77.8%

^1^ Proportion of athletes who met or exceeded the total and meal-specific dietary protein amounts recommended by the UEFA expert panel.

**Table 4:** Absolute and relative mean intakes of protein at meals ^1^.

	Men	Women
Breakfast protein (g)	23.25 ± 15.19	16.20 ± 6.25
Breakfast protein (g/kg BW)	0.33 ± 0.23	0.26 ± 0.11
Lunch protein (g)	42.54 ± 24.79	24.42 ± 10.28
Lunch protein (g/kg BW)	0.60 ± 0.39	0.38 ± 0.16
Dinner protein (g)	51.77 ± 20.51	31.11 ± 11.60
Dinner protein (g/kg BW)	0.71 ± 0.26	0.49 ± 0.16

^1^ Overview of meal-specific protein intake characteristics among men and women. Data are presented as means ± SD.

## Data Availability

Data are available upon request from corresponding authors.
